# A predictive risk score to diagnose hypocalcemia after parathyroidectomy in patients with secondary hyperparathyroidism: a 22-year retrospective cohort study

**DOI:** 10.1038/s41598-022-13880-0

**Published:** 2022-06-09

**Authors:** Mattabhorn Phimphilai, Suchada Inya, Worapaka Manosroi

**Affiliations:** 1 Division of Endocrinology, Department of Internal Medicine, Faculty of Medicine, Chiang Mai University, Chiang Mai, Thailand; 2 Department of Internal Medicine, Faculty of Medicine, Chiang Mai University, Chiang Mai, Thailand

**Keywords:** Endocrinology, Nephrology

## Abstract

Hypocalcemia is a common complication found in patients with secondary hyperparathyroidism (SHPT) who undergo parathyroidectomy. This study aimed to construct a predictive risk score for the occurrence of hypocalcemia after parathyroidectomy in patients with SHPT who underwent chronic renal replacement therapy (RRT). This 22-year retrospective cohort study enrolled 179 patients with SHPT who had their first parathyroidectomy. Eighty-two percent of patients developed hypocalcemia within 16.9 (95% CI 14.5–19.5) h after parathyroidectomy. This study demonstrated four factors as independent risk factors for post-parathyroidectomy hypocalcemia, including duration of RRT, preoperative serum phosphate, preoperative serum alkaline phosphatase (ALP) and mean difference of serum intact parathyroid hormone (iPTH). By using logistic regression analysis, this study demonstrated cut-off points for these four risk factors for the diagnosis of hypocalcemia after parathyroidectomy: 5 years for the duration of RRT, 5 mg/dL for serum phosphate, 387 U/L for serum ALP, and 97% for the mean difference of serum iPTH. Finally, the predictive risk score was constructed by assigning a score of one to each factor. With a total score of at least 2, the proposed predictive risk score has an AuROC of 0.755 with a sensitivity of 78.2%, a specificity of 71.4%, and an accuracy of 76.9%.

## Introduction

Secondary hyperparathyroidism (SHPT) is a part of chronic kidney disease-mineral and bone disorder (CKD-MBD) that occurs as early as chronic kidney disease stage 2 and then worsens with a declining glomerular filtration rate (GFR)^1^. Approximately 56% of patients with chronic kidney disease stage 3 have SHPT, and higher than 80% of patients with end-stage kidney diseases develop SHPT^1^. SHPT is characterized by an elevation of serum parathyroid hormone (PTH) and parathyroid gland hyperplasia. It is caused by the alterations of minerals and hormones due to the decline of GFR, including an increase in serum phosphate and fibroblast growth factor 23 (FGF23) but a decline in serum calcium and calcitriol^1,2^. SHPT is associated with an increased risk of bone fractures and vascular calcification^3^, entailing increased risks of morbidity and mortality in patients with CKD^4,5^.

SHPT is primarily treated with medications, including phosphate binders, vitamin D analogs, and calcimimetics^6,7^. According to the effectiveness of newer calcimimetics^7^, surgical treatment is reserved only for patients with inadequate response or intolerance to medications^6,8^, for example, cases with refractory hyperphosphatemia or persistent elevated PTH level of higher than 800 pg/mL, cases with calciphylaxis, and cases with complications of SHPT^6,8,9^. Parathyroidectomy has been shown to provide benefits for all-cause mortality and cardiovascular mortality in SHPT^10,11^. Subtotal parathyroidectomy (SPTX), total parathyroidectomy (TPTX), or total parathyroidectomy with autotransplantation (TPTX/AT) are the surgical options for parathyroidectomy. However, there is no definite conclusion for the most appropriate surgical option in patients with SHPT^10,12–14^.

Hypocalcemia and severe hypocalcemia, with serum calcium of lower than 8.5 mg/dL and 7.6 mg/dL, respectively, are common complications that occur after parathyroidectomy in patients with SHPT. The prevalence of post-parathyroidectomy hypocalcemia ranged from 27 to 82%, as documented in multiple previous reports^15–26^. Multiple studies demonstrated independent risk factors, including patient factors, preoperative biochemical parameters, and intraoperative factors, for severe hypocalcemia after parathyroidectomy^16–22,24–28^. However, to date, few studies have focused on the risk factors influencing overall hypocalcemia after parathyroidectomy^15,23,29^. Multiple factors were demonstrated as independent risk factors for overall post-parathyroidectomy hypocalcemia, including age^15,29^, preoperative serum calcium^15,29^, preoperative serum alkaline phosphatase (ALP)^15,23,29^, the preoperative serum PTH^29^, and weight of excised parathyroid glands^15^. Even though several studies demonstrated multiple independent risk factors for overall post-parathyroidectomy hypocalcemia^15,23,29^, none of those studies demonstrated the cut-off points of their identified risk factors, and so the predictive risk score for post-parathyroidectomy hypocalcemia have never been constructed for application use in clinical practice. Therefore, this study aimed to construct a predictive risk score from simple patient factors and routine biochemical parameters to determine the risk of overall post-parathyroidectomy hypocalcemia in patients with SHPT who underwent chronic RRT.

## Results

### Baseline characteristics and univariable analysis of factors associated with hypocalcemia after parathyroidectomy

This study enrolled 179 patients with SHPT who underwent chronic RRT. One hundred and forty-six patients (81.6%) had chronic hemodialysis as a mode of RRT, while 33 patients (18.4%) received continuous ambulatory peritoneal dialysis (CAPD) as a mode of RRT. The duration of RRT was 7.3 (95% CI 2.6–2.8) years before parathyroidectomy. Preoperative serum corrected calcium and phosphate were 9.9 (95% CI 9.7–10.0) mg/dL and 5.3 (95% CI 5.1–5.6) mg/dL, respectively. Ninety-five patients (53.1%) were preoperatively treated with oral calcium. Seventy-six patients (42.5%) were preoperatively treated with either vitamin D analogs or calcitriol. Of these, most patients (91.7%) received alfacalcidol at a daily dosage of 0.4 µg (95% CI 0.3–0.4), while 8.3% of patients received calcitriol at a dosage of 0.9 µg (95% CI 0.3–1.6). None of the patients developed hypocalcemia before parathyroidectomy. Only 17 patients (9.5%) were treated with cinacalcet, a calcimimetic, at a daily dosage of 35.3 mg (95% CI 28.8–41.8). Preoperative serum ALP and PTH levels were 318.4 U/L (95% CI 275.8–367.6) and 1926.8 pg/mL (95% CI 1796.8–2066.21), respectively. Almost 40% (71/179) of patients had abnormal radiologic findings consistent with osteitis fibrosa cystica on conventional bone survey preoperatively. Most patients had a low risk of fractures, as determined by the Fracture Risk Assessment Tool (FRAX^®^) using the Thailand database^30^. The 10-year probability for hip fractures and major osteoporotic fractures were 0.2% (95% CI 0.1–0.2) and 1.4% (95% CI 1.3–1.6). Consistent with the low probability of fractures, only six patients experienced non-traumatic fractures.

**Figure 1:**
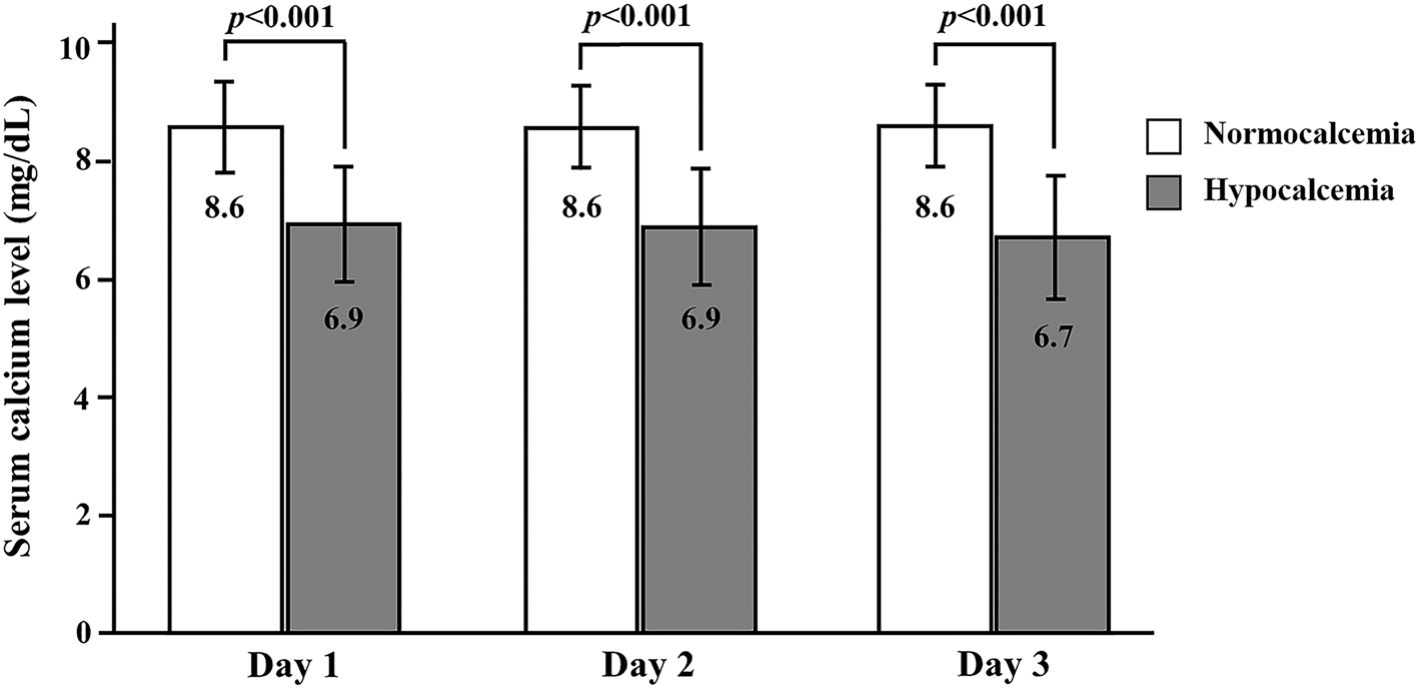
Serum calcium level during the first 3-day postparathyroidectmy period. The bar graph showed a comparison of serum calcium level between patients who developed hypocalcemia after parathyroidectomy and patients who remained normocalcemia during the first 3 days after parathyroidectomy (mean ± SD). The levels of serum calcium at the first (7.0 ± 1.0 mg/dL vs. 8.6 ± 0.8 mg/dL, *p* < 0.001), second (6.9 ± 0.9 mg/dL vs. 8.6 ± 0.7 mg/dL, *p* < 0.001) and third day (6.7 ± 1.0 mg/dL vs. 8.6 ± 0.7 mg/dL, *p* < 0.001) after parathyroidectomy were significantly lower in patients who developed hypocalcemia than those patients who remained normocalcemia.

Most of the patients (79.3%) had total parathyroidectomy with autotransplantation (TPTX/AT) as a surgical option. Of these, 81% and 14.1% had parathyroid tissue reimplanted within the brachioradialis and sternocleidomastoid muscles, respectively. A minority of patients, at 12.3% and 8.5%, had subtotal parathyroidectomy (SPTX) and total parathyroidectomy (TPTX), respectively. All had parathyroidectomy done as indicated by uncontrolled SHPT despite medical therapy. Almost all patients (93.3%) had pathologically confirmed hyperplasia of multiple parathyroid glands, consistent with the preoperative diagnosis of SHPT. The mean operative time was 2.7 (95% CI 2.6–2.8) h. The mean difference of serum parathyroid hormone was 89.9% (95% CI 87.8–92.0) of the preoperative level at 24 h after parathyroidectomy. Serum calcium was routinely monitored every 4–6 h within the first 72 h after parathyroidectomy. This study showed that 82.1% (147/179) of patients developed hypocalcemia after parathyroidectomy, while 64.2% (115/179) developed severe hypocalcemia requiring intravenous calcium. The mean duration for the occurrence of hypocalcemia was 16.9 (95% CI 14.5–19.5) h. The levels of serum calcium (mean ± SD) on the first (7.0 ± 1.0 mg/dL vs. 8.6 ± 0.8 mg/dL, *p* < 0.001), second (6.9 ± 0.9 mg/dL vs. 8.6 ± 0.7 mg/dL, *p* < 0.001) and third days (6.7 ± 1.0 mg/dL vs. 8.6 ± 0.7 mg/dL, *p* < 0.001) after parathyroidectomy were significantly lower in patients who developed hypocalcemia than in those who remained normocalcemia (Fig. 1).

Univariate analysis demonstrated that mode of RRT, duration of RRT, and surgical procedure were significant differences between groups with hypocalcemia and normocalcemia after parathyroidectomy (Table 1). A longer duration of RRT is associated with a higher risk of postoperative hypocalcemia, whereas CAPD and subtotal parathyroidectomy are associated with a smaller risk of postoperative hypocalcemia (Table 1). In terms of preoperative biochemical parameters, higher serum phosphate, serum ALP, and serum iPTH were significantly associated with postoperative hypocalcemia (Table 1). In terms of postoperative biochemical parameters, the risk of postoperative hypocalcemia increased with higher serum phosphate, lower serum iPTH, and a larger serum iPTH decrement after parathyroidectomy (Table 1).

**Table 1:** Clinical characteristics of parathyroidectomy patients.

Characteristics	Normocalcemia (n = 32)	Hypocalcemia (n = 147)	OR	95% CI	*p* value
Age (years)	45.5 (41.3–49.8)	44.5 (42.4–46.5)	0.99	0.96–1.02	0.657
**Gender, n (%)**					
Female	19 (59.4)	80 (54.4)	Ref		
Male	13 (40.6)	67 (45.6)	1.22	0.56–2.66	0.610
**Concurrent illness, n (%)**					
Diabetes	3 (9.4)	12 (8.2)	0.86	0.23–3.24	0.823
Hypertension	21 (65.6)	100 (68.0)	1.11	0.50–2.50	0.793
Hyperlipidemia	8 (25.0)	38 (25.9)	1.05	0.43–2.52	0.921
Gout	6 (18.8)	21 (14.3)	0.72	0.27–1.96	0.524
Liver diseases	1 (3.1)	16 (10.9)	3.79	0.48–29.64	0.205
**Renal replacement therapy (RRT), n (%)**^**#**^					
Hemodialysis	21 (65.6)	124 (84.4)	Ref		
CAPD	10 (31.3)	23 (15.6)	0.39	0.16–0.93	0.035
Duration of RRT (years)^#^	5.8 (4.9–6.6)	7.6 (7.0–8.3)	1.17	1.03–1.32	0.015
**Preoperative therapy**					
Calcium, (%)	46.9	54.4	1.35	0.63–2.91	0.439
Vitamin D, (%)	43.8	42.2	0.94	0.43–2.03	0.870
**Preoperative investigation**					
Serum corrected calcium (mg/dL)	10.1 (9.7–10.4)	9.8 (9.7–10.0)	0.76	0.51–1.13	0.180
Serum phosphate (mg/dL)	4.7 (4.1–5.3)	5.5 (5.2–5.8)	1.31	1.03–1.66	0.029
Serum alkaline phosphatase (U/L)	188.2 (135.5–261.4)^†^	358.0 (306.6–418.0)^†^	1.00	1.00–1.00	0.041
Serum parathyroid hormone (pg/mL)	1465.9 (1166.5–1842.1)^†^	2045.0 (1912.7–2186.4)^†^	1.00	1.00–1.00	0.029
Radiologic bone change (n, %)	13 (40.6)	58 (39.5)	0.95	0.44–2.08	0.902
**Surgical procedure, n (%)**					
Subtotal PTX*	9 (28.1)	13 (8.8)	Ref.		
Total PTX*	4 (12.5)	11 (7.5)	1.90	0.46–7.92	0.376
Total PTX* with autotransplantation	19 (59.4)	123 (83.6)	4.48	1.69–11.91	0.003
Operative period (hours)	2.6 (2.3–2.9)	2.7 (2.6–2.8)	1.15	0.69–1.90	0.589
Parathyroid volume removal (grams)	3.1 (2.3–4.2)^†^	4.2 (3.7–4.8)^†^	1.11	0.98–1.25	0.100
**Postoperative investigation**					
Serum corrected calcium (mg/dL)	8.6 (8.4–8.8)	6.9 (6.8–7.0)	0.02	0.00–0.08	< 0.001
Serum phosphate (mg/dL)	0.8 (0.4–1.3)^†^	1.8 (1.5–2.1)^†^	1.53	1.12–2.10	0.008
Serum parathyroid hormone (PTH) (pg/mL)	124.9 (73.1–213.3)^†^	80.7 (65.5–99.3)^†^	0.999	0.997–1.000	0.020
Means difference of serum PTH (%)	80.6 (72.5–88.8)	92.0 (90.3–93.7)	1.05	1.02–1.08	< 0.001

^#^Analyzed data from 145 patients in hemodialysis group and 33 patients in CAPD group.

*PTX parathyroidectomy, ^†^data presented as Geometric mean (95% CI).

### Multivariable analysis for factors associated with hypocalcemia after parathyroidectomy

All factors in univariate analysis were applied in multivariate analysis to determine independent risk factors for postoperative hypocalcemia. Following multivariate regression analysis, preoperative serum phosphate and ALP, and the mean difference in serum iPTH between before and after parathyroidectomy, were associated with a significantly increased risk of postoperative hypocalcemia. Every 1 mg/dL increase in preoperative serum phosphate and every 10 U/L increase in preoperative serum ALP increased the risk of postoperative hypocalcemia by 48.5% (AOR 1.485, 95% CI 1.089–2.025) and 2% (AOR 1.002, 95% CI 1.001–1.004), respectively (Table 2). Every 10% difference in serum iPTH before and after parathyroidectomy was associated with a 6.9% increase in the risk of postoperative hypocalcemia (AOR 1.069, 95% CI 1.025–1.114) (Table 2). Longer durations of RRT tended to be associated with postoperative hypocalcemia. Each year of RRT increased the risk of post-parathyroidectomy hypocalcemia by 18.1% (AOR 1.181, 95% CI 0.993–1.407), but this did not reach statistical significance (Table 2).

**Table 2:** Multivariable regression analysis of predictive factors for post-parathyroidectomy hypocalcemia.

Factor	Unit	AOR	95% CI	*p* value
Duration of RRT	1 year	1.181	0.993–1.407	0.061
Preoperative serum phosphate	1 mg/dL	1.485	1.089–2.025	0.012
Preoperative serum alkaline phosphatase	10 U/L	1.002	1.001–1.004	0.006
Means difference of serum PTH	10%	1.069	1.025–1.114	0.002

### Predictive risk score to diagnose hypocalcemia after parathyroidectomy

**Figure 2:**
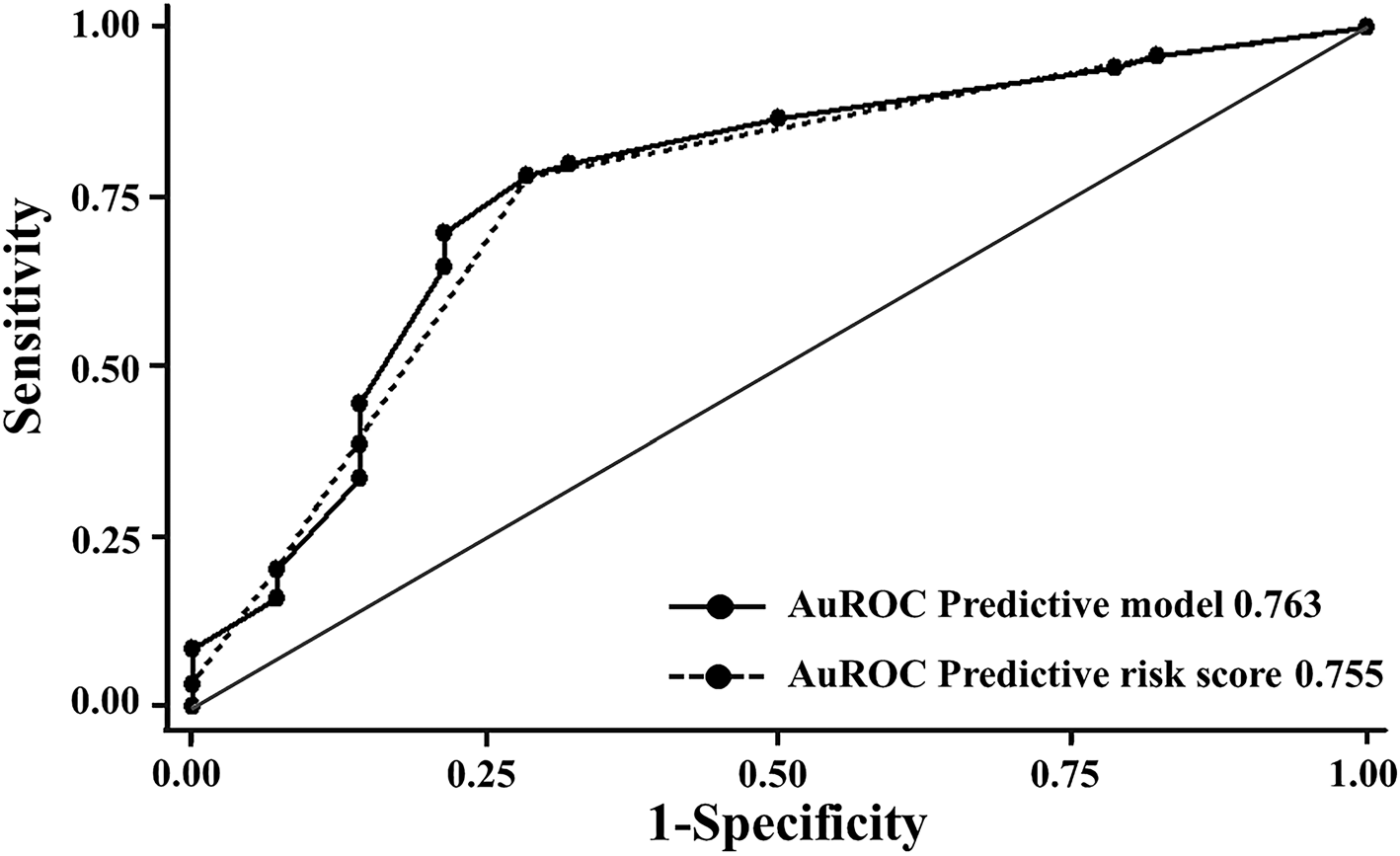
ROC and Area under ROC (AuROC) for the prediction of hypocalcemia after parathyroidectomy. Area under the receiver operating characteristic (AuROC) curve of hypocalcemia after parathyroidectomy predicted by predictive model (solid line), predictive risk score (dash line), and 50% chance prediction (diagonal line).

Logistic regression analysis was next performed to identify the cut-off points for each risk factor identified in multivariate analysis and then to create a predictive risk score for the diagnosis of post-parathyroidectomy hypocalcemia by using those cut-off levels. By categorizing each factor into two groups, the duration of RRT at the cut-off point of 5 years showed an AOR of 3.47 (95% CI 1.37–8.77) with a β-coefficient of 1.244 (Table 3). Either preoperative serum phosphate at the cut-off level of 5 mg/dL or the mean difference of serum iPTH between pre-and post-parathyroidectomy at the cut-off point of 97% showed an AOR of 3.09 (95% CI 1.02–9.31) with a β-coefficient of 1.128 (Table 3). Preoperative serum ALP at the cut-off level of 387 U/L showed an AOR of 3.83 (95% CI 1.25–11.77) with a β-coefficient of 1.343 (Table 3). The area under the receiver operating characteristic (AuROC) curve was 0.763 (Fig. 2). Finally, a score of one was assigned to each factor at its specific cut-off point, and any combination of each factor leading to a total score of at least two points would categorize patients into a high-risk group for developing hypocalcemia after parathyroidectomy (Table 4). The diagnostic performance of the predictive risk score for hypocalcemia after parathyroidectomy at the threshold of two was 78.2% for sensitivity, 71.4% for specificity, 76.9% for accuracy, 92.1% for positive predictive value (PPV), and 43.5% for negative predictive value (NPV). The AuROC of the predictive risk score was 0.755, which was comparable to the AuROC from the predictive model (Fig. 2).

**Table 3:** Multivariate logistic regression analysis with coefficient values of each factor and their assigned score.

Predictive factors	AOR	95% CI for AOR	β-coefficient	Item score	Assigned score	*p* value
**Duration of RRT**						
≤ 5 years	Ref.			0	0	
> 5 years	3.47	1.37–8.77	1.244	1.103	1	0.009
**Preoperative serum phosphate**						
< 5 mg/dL	Ref.			0	0	
≥ 5 mg/dL	3.09	1.19–8.04	1.128	1.001	1	0.021
**Preoperative serum alkaline phosphatase**						
< 387 U/L	Ref.			0	0	
≥ 387 U/L	3.83	1.25–11.77	1.343	1.191	1	0.019
**Means difference of serum parathyroid hormone**						
< 97%	Ref			0	0	
≥ 97%	3.09	1.02–9.31	1.128	1.000	1	0.045

**Table 4:** Risk score for the diagnosis of post-parathyroidectomy hypocalcemia.

Criteria	Score
**Duration of RRT > 5 years**	
Yes	1
No	0
**Preoperative serum phosphate ≥ 5 mg/dL**	
Yes	1
No	0
**Preoperative serum alkaline phosphatase ≥ 387 U/L**	
Yes	1
No	0
**Means difference of serum parathyroid hormone ≥ 97%**	
Yes	1
No	0
Total	4
High risk for hypocalcemia if the total score is ≥ 2	

## Discussion

This study demonstrated four factors as independent risk factors for overall post-parathyroidectomy hypocalcemia in patients with SHPT who underwent chronic RRT. Three of the identified factors had never been shown as predictive factors for post-parathyroidectomy hypocalcemia, including duration of RRT, preoperative serum phosphate, and mean difference in serum iPTH between pre-and post-parathyroidectomy. This study also demonstrated cut-off points for these four risk factors for the diagnosis of post-parathyroidectomy hypocalcemia: 5 years for the duration of RRT; 5 mg/dL for serum phosphate; 387 U/L for serum ALP; and 97% for the mean difference of serum iPTH. In addition, this study was the first to construct a predictive risk score for the occurrence of overall hypocalcemia after parathyroidectomy in cases with SHPT. By assigning a score of one to each independent risk factor, a total score of at least two had a sensitivity of 78.2%, a specificity of 71.4%, and an accuracy of 76.9% for predicting post-parathyroidectomy hypocalcemia.

This long-term retrospective cohort study, performed in a single center, demonstrated overall hypocalcemia of 82.1% and severe hypocalcemia of 64.2% after parathyroidectomy. This high incidence of hypocalcemia could be associated with the extremely high level of serum iPTH at 1926.8 pg/mL (95% CI 1796.8–2066.21) shown in this study. However, this high incidence of hypocalcemia and extremely high level of iPTH were in the same range as documented in previous reports^15,20–22,24,28^. This study demonstrated four factors as independent risk factors for overall hypocalcemia after parathyroidectomy in SHPT. One factor, preoperative serum ALP, was consistent with other previous reports^15,23,29^. In terms of using preoperative serum ALP as a predictor for overall hypocalcemia, this study is the first to propose a cut-off point for application in clinical practice. Our proposed cut-off level was 387 U/L which is positioned between two cut-off points proposed by Kritmetapak and colleagues^22^ (420 U/L) and Zou and colleagues^26^ (277 U/L) for the prediction of severe hypocalcemia after parathyroidectomy in cases with SHPT. Another three factors, including the duration of RRT, preoperative serum phosphate, and mean difference of serum iPTH, have never been identified in previous reports as independent risk factors for overall hypocalcemia after parathyroidectomy.

In terms of serum phosphate level, preoperative serum phosphate was shown by Tsai and colleagues as a risk factor for severe hypocalcemia after parathyroidectomy^17^. Since the retention of phosphate due to a decline in glomerular filtration rate (GFR) is the main pathophysiology of parathyroid gland hyperplasia and parathyroid hormone stimulation in patients with chronic kidney disease^1,2^, a higher level of preoperative serum phosphate level would reasonably be linked to a higher stimulation of PTH secretion and higher bone resorption and then higher skeletal mineral uptake after parathyroidectomy. Since serum phosphate is recommended to be maintained at a normal range in patients with end-stage kidney disease undergoing chronic RRT^6^, our proposed cut-off point of 5 mg/dL in this study was aligned with the standard clinical practice guidelines.

Although preoperative serum iPTH had previously been shown to be an independent risk factor for both overall hypocalcemia^29^ and severe hypocalcemia^17,19,24,25,27^ after parathyroidectomy in SHPT, our study found that preoperative serum iPTH was only associated with postoperative hypocalcemia in the univariate analysis and not in the multivariate analysis. The mean difference in serum iPTH between pre- and post-parathyroidectomy was found to be an independent risk factor for overall hypocalcemia after parathyroidectomy in our study. Our study demonstrated this independent risk factor as a novel factor for overall hypocalcemia after parathyroidectomy. The greater the difference in serum iPTH, the greater the volume of parathyroid tissue that may be removed during parathyroidectomy. Torer and colleagues^15^ demonstrated that the quantity of excised parathyroid tissue was an independent risk factor for overall hypocalcemia after parathyroidectomy in SHPT. Wang and colleagues^24^ also demonstrated the quantity of excised parathyroid tissue as an independent risk factor of severe hypocalcemia after parathyroidectomy in SHPT. Since the success of parathyroidectomy is defined by the sustained reduction of parathyroid hormone after parathyroidectomy, pre-and postoperative serum iPTH measurement is recommended for all cases with SHPT who underwent parathyroidectomy to determine the outcome of the operation. Therefore, the mean difference in serum iPTH could be acquired from routine clinical practice, which was easier than obtaining the information involving the quantity of excised parathyroid glands. Even though the reduction of intraoperative serum iPTH at least 50% of the preoperative level is well established as a predictor for disease remission in primary hyperparathyroidism, the intraoperative serum iPTH monitoring in SHPT remains to be clarified for its benefits, as it is in cases with primary hyperparathyroidism^9,31,32^. Nevertheless, a decrease in serum iPTH at least 70% of the preoperative level is acceptable to predict the success of the parathyroidectomy in cases with SHPT^31,32^. Since the criteria of serum iPTH reduction for determining the success of the parathyroidectomy in SHPT ranged from 50 to 90% of preoperative levels^31^, our mean difference in serum iPTH at the cut-off threshold of 97% was reasonable to indicate the excessive reduction of serum iPTH.

The duration of RRT with a cut-off point of 5 years was a novel factor found in this study. The longer duration of RRT may indicate the longer duration of SHPT and other metabolic alterations involving CKD-MBD. Other factors previously identified as independent risk factors for overall hypocalcemia, including age and preoperative serum calcium^15,29^, were not demonstrated as risk factors in our study. Age and preoperative serum calcium did not show any difference in both the hypocalcemic and normocalcemic groups.

This study was the first to construct a predictive risk score for the occurrence of overall hypocalcemia after parathyroidectomy in cases with SHPT. By using the proposed cut-off points of those four factors, the predictive risk score was constructed by assigning a score of one to each factor. With a threshold of two, our proposed predictive risk score has an AuROC of 0.755, which is comparable to the AuROC of our predictive model (0.763). In addition, with a threshold of two, our predictive risk score had a sensitivity of 78.2%, a specificity of 71.4%, and an accuracy of 76.9%, indicating a good potential for predicting overall hypocalcemia after parathyroidectomy in SHPT.

This study had multiple strength points involving the originality of our findings. First, this study identified four independent risk factors for overall hypocalcemia that could be linked to the pathophysiology of SPHT. Those identified factors were composed of three factors that have never been reported for overall hypocalcemia after parathyroidectomy in SHPT. Furthermore, all the identified factors were simple factors that could be acquired from routine clinical practice. Second, this study was the first to propose a cut-off point of each risk factor for further application in a clinic. Third, this study was the first to construct a simple predictive risk score for the prediction of overall hypocalcemia after parathyroidectomy in SHPT and this predictive risk score showed good performance in predicting the occurrence of hypocalcemia after parathyroidectomy. However, the results should be interpreted with caution due to some limitations. First, this study was a retrospective study. As a result of the nature of this type of study, unexpected confounding factors and bias may occur. Some factors that were not determined in this study may influence the results. In addition, some information could not be completely retrieved, which may influence the results of the study; for example, the quantity of parathyroid gland left in situ in SPTX and the quantity of reimplanted parathyroid tissue in cases with TPTX/AT. Second, this study was a single-center study. Therefore, it may limit the application of this use in patients with different backgrounds.

In conclusion, this study demonstrated four independent risk factors for overall post-parathyroidectomy hypocalcemia in patients with SHPT who underwent chronic RRT. In addition, this study demonstrated cut-off points for these four risk factors: 5 years for the duration of RRT; 5 mg/dL for serum phosphate; 387 U/L for serum ALP; and 97% for the mean difference of serum iPTH. By using the proposed cut-off points of these four factors, the predictive risk score was constructed by assigning a score of one to each factor. With a total score of at least two, the proposed predictive risk score showed good diagnostic performance for overall hypocalcemia after parathyroidectomy. Since this risk score was generated by using parameters acquired in routine clinical practice, this predictive risk score could be a simple tool for clinicians to use for identifying cases at risk for post-parathyroidectomy hypocalcemia and providing them with guidance for closely monitoring serum calcium in those high-risk cases to prevent severe hypocalcemia after parathyroidectomy.

## Methods

A 22-year retrospective cohort study was conducted at the Internal Medicine Outpatient Department unit of Maharaj Nakorn Chiangmai Hospital, Thailand. The study was approved by the Institutional Board Review of the Faculty of Medicine Chiang Mai University. The ethical code is EXEMPTION-6523/2019; informed consent was exempted by the Research Ethics Committee. All methods were performed in accordance with the guidelines and regulations of our institute, as well as with the Declaration of Helsinki. Inclusion criteria were adult patients aged 18–70 with SHPT who underwent chronic RRT who had parathyroidectomy for the first time during January 1997–July 2019. Exclusion criteria were as follows: patients with incomplete perioperative biochemical measurements, including serum calcium, phosphate, ALP, intact parathyroid hormone (iPTH); patients with incomplete information of the surgical procedure and pathological reports; patients who did not regularly follow-up at the Internal Medicine Outpatient Department Unit of Maharaj Nakorn Chiang Mai Hospital, Thailand.

### Definitions

SPHT is defined as a compensatory hypersecretion of parathyroid hormone and hyperplasia of the parathyroid glands due to abnormalities in calcium homeostatsis. Since the Kidney Disease: Improving Global Outcomes (KDIGO)^6,8^ recommends optimizing serum parathyroid hormone levels at two-to-nine folds of the upper normal limit in patients with chronic kidney stage 5 undergoing chronic RRT (CKD5D), SPHT was defined as a serum parathyroid hormone level greater than 585 pg/mL (normal reference range 15–65 pg/mL) in CKD5D patients with no spontaneous hypercalcemia in this study. CKD5D patients who developed both hyperparathyroidism and spontaneous hypercalcemia were defined as having tertiary hyperparathyroidism but not SPHT. Hypocalcemia was defined as serum corrected calcium lower than 8.5 mg/dL that lasted more than 3 days. Serum corrected calcium was calculated if serum albumin was lower than 4 g/L by using the following formula—serum corrected calcium = serum measured calcium + [(4.0 − serum albumin) × 0.8]. Severe hypocalcemia was defined as serum corrected calcium of lower than 7.6 mg/dL.

### Predictive variables

Demographic, clinical, and biochemical data were obtained from electronic medical records. Age, gender, mode and duration of RRT, indication for parathyroidectomy, surgical procedure and pathological report, postoperative symptoms of tetany, were retrieved. Preoperative and postoperative biochemical parameters, including serum calcium, serum phosphate, serum albumin, serum ALP, serum iPTH, were also collected. Serum calcium measured preoperatively and the first 72 h after parathyroidectomy were retrieved. Serum iPTH measured at 24 h postoperatively were collected. All biochemical parameters were assessed using standardized procedures at the central laboratory of the Faculty of Medicine, Chiang Mai University. Bone change consistent with osteitis fibrosa cystica was evaluated by conventional bone survey radiography. Fracture risk estimation was estimated from the Fracture Risk Assessment Tool (FRAX^®^) using the Thailand database^30^.

### Outcome variables

The outcome of parathyroidectomy was categorized into two groups: hypocalcemia and normocalcemia.

### Statistical analysis

The analysis was performed by STATA program version 14.2. The statistical significance level was set as two-tailed with a *p* value of < 0.05. Categorical variables are reported as counts or percentages; for normally distributed continuous variables, means and 95% confidence interval (95% CI) are presented unless indicated otherwise. For non-normally-distributed continuous variables, geometric means and 95% CI are reported. Univariable and multivariable regression analyses were conducted by logistic regression analysis and are reported as odds ratio (OR) or adjusted odds ratio (AOR) and 95% CI, respectively. The final predictive model was developed by stepwise backward selection by removing the factors with *p* value > 0.2. To create item score, the adjusted odds ratio was converted to ß-coefficient. The ß-coefficient of each factor was divided by the smallest ß-coefficient in the final model and rounded to the nearest 0.5. The total score was calculated by combining all the item scores and divided into high- and low-risk groups. Sensitivity, specificity, positive predictive value (PPV), negative predictive value (NPV) and accuracy were calculated for each risk level. The cut-off point for the risk levels was retrieved from the level that yielded the highest sensitivity, specificity, and accuracy. The distinction between the predictive scoring system and predictive model accuracy was demonstrated by AuROC. The sample size was calculated based on the rule of thumb which is the sample size would require at least ten events for each candidate predictive factor that will be included in the prediction model. Therefore, at least 40 patients for four predictive factors should be included.

## Data Availability

All data generated or analysed during this study are included in this published article.
